# Novel Strategy to Combat Antibiotic Resistance: A Sight into the Combination of CRISPR/Cas9 and Nanoparticles

**DOI:** 10.3390/pharmaceutics13030352

**Published:** 2021-03-08

**Authors:** Fen Wan, Mohamed S. Draz, Mengjie Gu, Wei Yu, Zhi Ruan, Qixia Luo

**Affiliations:** 1 State Key Laboratory for Diagnosis and Treatment of Infectious Diseases, Collaborative Innovation Center for Diagnosis and Treatment of Infectious Diseases, The First Affiliated Hospital, Zhejiang University School of Medicine, Hangzhou 310003, China; fenw@hmc.edu.cn (F.W.); wyu@zju.edu.cn (W.Y.); 2 College of Laboratory Medicine, Hangzhou Medical College, Hangzhou 310003, China; 3 Department of Medicine, Case Western Reserve University School of Medicine, Cleveland, OH 44106, USA; mdraz@gmwgroup.harvard.edu; 4 Department of Electrical and Computer Engineering, Tufts University, Medford, MA 02155, USA; 5 Department of Mechanical Engineering, Rice University, Houston, TX 77005, USA; 6 Department of Chemistry and Chemical Biology, Harvard University, Cambridge, MA 02138, USA; 7 Key Lab of Drug Clinical Research and Evaluation Technology of Zhejiang Province, The First Affiliated Hospital, Zhejiang University School of Medicine, Hangzhou 310003, China; mengjie_gu@u.nus.edu; 8 Department of Clinical Laboratory, Sir Run Run Shaw Hospital, Zhejiang University School of Medicine, Hangzhou 310016, China

**Keywords:** CRISPR/Cas9, nanoparticle systems, antibiotic resistance, delivery

## Abstract

Antibiotic resistance is a significant crisis that threatens human health and safety worldwide. There is an urgent need for new strategies to control multidrug-resistant (MDR) bacterial infections. The latest breakthrough in gene-editing tools based on CRISPR/Cas9 has potential application in combating MDR bacterial infections because of their high targeting ability to specifically disrupt the drug resistance genes that microbes use for infection or to kill the pathogen directly. Despite the potential that CRISPR/Cas9 showed, its further utilization has been hampered by undesirable delivery efficiency in vivo. Nanotechnology offers an alternative way to overcome the shortcomings of traditional delivery methods of therapeutic agents. Advances in nanotechnology can improve the efficacy and safety of CRISPR/Cas9 components by using customized nanoparticle delivery systems. The combination of CRISPR/Cas9 and nanotechnology has the potential to open new avenues in the therapy of MDR bacterial infections. This review describes the recent advances related to CRISPR/Cas9 and nanoparticles for antimicrobial therapy and gene delivery, including the improvement in the packaging and localizing efficiency of the CRISPR/Cas9 components in the NP (nanoparticle)/CRISPR system. We pay particular attention to the strengths and limitations of the nanotechnology-based CRISPR/Cas9 delivery system to fight nosocomial pathogens.We highlight the need for more scientific research to explore the combinatorial efficacy of various nanoparticles and CRISPR technology to control and prevent antimicrobial resistance.

## 1. Introduction

Antibiotics have protected millions of people from bacterial infections through their remarkable ability to kill or inhibit the growth of bacterial pathogens [1,2]. Antibiotic resistance occurs when bacteria change over time and no longer respond to the available antimicrobial agents, making it harder to treat and increasing the risk of disease spread, severe illness, and death [2]. The recent increase in antibiotic resistance is dramatic and has rendered most of the available antibiotics less effective [3,4,5]. The World Health Organization (WHO, Geneva, Switzerland) has declared that antibiotic resistance is one of the top ten global public health threats facing humanity. In addition to death and disability, antibiotic has a significant social and economic impact caused by prolonged illness, extended hospitalization and healthcare, the need for more expensive medicine, which weigh most heavily on lower- and middle-income countries [6].

Misuse and overuse of antibiotics are the main driving force behind the development of multidrug resistance in bacteria [2,7]. Multidrug-resistant (MDR) bacteria succeed to acquire resistance that inactivate most of the antibiotics (including those that are considered the last resort of defense) through sequential genetic mutations and horizontal transfer of mobile genetic elements, increasing the importance for developing more effective antimicrobial agents [8]. In addition, traditional antibiotics indiscriminately kill beneficial bacteria, and deleteriously affect the commensal human microbiota [9]. Together, this highlights the need for new approaches that adopt different bactericidal mechanisms to avoid drug resistance and provide the capability to only target harmful bacteria and with minimal effect on the patient and other beneficial bacteria. The clustered regularly interspaced short palindromic repeats (CRISPR) CRISPR-associated protein (Cas) system can provide versatile and promising tools against the growing challenge of multidrug resistance prevalence [10]. Among the different types of CRISPR-Cas systems, the CRISPR-Cas9 system is the most widely applied in gene editing [11]. By designing guide RNAs, the CRISPR-Cas9 system can kill targeted bacterial species possessing specific sequences in the involved bacterial community or destroy their antibiotic resistance genes, resensitizing them to antibiotics [12]. Although the CRISPR-Cas9 system is a powerful and efficient tool to address the myriad of multidrug-resistant microbial infections, its delivery has become the major limitation for therapeutic applications [13]. Currently, both virus-based and nonviral gene delivery systems have been studied as delivery platforms for the CRISPR-Cas system [14]. However, compared to virus-based delivery, nonviral delivery systems such as nanoparticles might have more potential for future use, as it overcomes multiple disadvantages [15], such as toxicity and immunogenicity, previously reported for the use of viral vectors in gene delivery.

In this review, we aim to summarize the role of the CRISPR-Cas9 system in combating MDR bacteria, and we highlight the potential of nanoparticles to enhance the delivery of the CRISPR-Cas9 system.

## 2. CRISPR-Cas System

### 2.1. The CRISPR-Cas System Protects Bacteria from Phage Invasion

The CRISPR-Cas system, derived from the adaptive immune system of prokaryotes, has been found in approximately 50% of bacterial genomes and 87% of archaea [16]. CRISPR-Cas system was first observed in 1987 when Ishino reported a repeat sequence of unknown function in the *Escherichia coli* K12 genome [17]. It was not until 2002 that the repeat sequence was named CRISPR [18]. Barrangou et al. conducted phage infection experiments and reported that CRISPR and its adjacent cas gene could protect bacteria from phage invasion [19]. The CRISPR-Cas systems are composed of a genetic locus, which contains a CRISPR array of repetitive sequences (repeats) interspaced by short stretches of non-repetitive sequences (spacers), and 6–20 genes encoding CRISPR-associated (cas) proteins [20]. In addition, the leader region is adjacent to the CRISPR array and is required to guide the spacers towards the right location [21]. Sequences of leaders and repeats interact together to direct the specificity of spacer integration [22]. Spacer sequences are known as protospacers and are derived from the genetic elements of invading phages and plasmids. The *cas* operon, which lies upstream of the CRISPR array and determines the system′s gene-editing efficiency, plays a critical role in the CRISPR system.

The CRISPR-Cas system in bacteria degrades foreign DNA fragments in three steps: adaptation, expression, and interference [23]. In the stage of transformation or acquisition, an approximately 30 bp spacer sequence is integrated into the CRISPR array by Cas1 and Cas2. The second stage is the expression of CRISPR RNA (crRNA), in which spacers of the CRISPR locus (pre-crRNA) are transcribed and processed into crRNA [23]. These pre-crRNAs are cleaved by specific endoribonucleases to yield short mature crRNAs. The crRNA contains the spacer at the 5′ end and a repeat at the 3′ end. Ultimately, in the interference stage, crRNA recognizes and forms a base pair specific to the foreign target sequence. This hybridization leads to the sequence-specific cleavage of the crRNA-foreign sequence complex by Cas nucleases upon the second infection. It is worth noting that a short-conserved sequence known as the protospacer adjacent motif PAM (2–5 bp), located in close proximity to the sequence identical to the spacer on the foreign DNA, is indispensable for targeted DNA selection and degradation [24].

### 2.2. The CRISPR-Cas9 System Applied to Genome Editing

**Figure 1:**
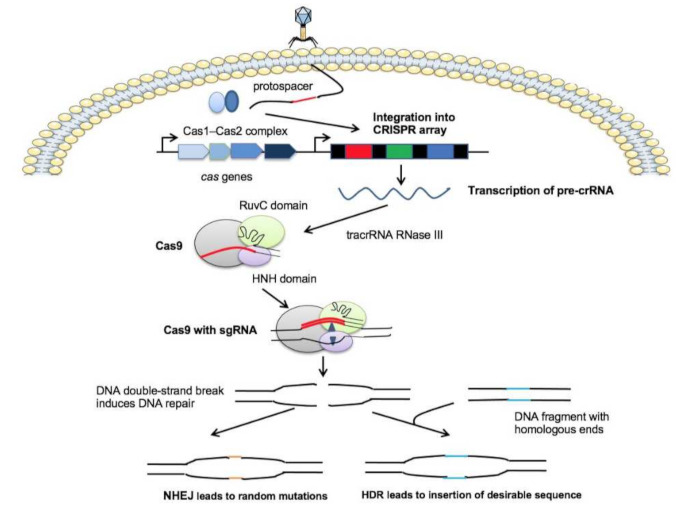
Molecular mechanism of the CRISPR-Cas 9 system. CRISPR-Cas systems are composed of a *cas* operon (blue arrows) and a CRISPR array which identical repeat sequences (black rectangles) that are interspersed by phage-derived spacers (colored rectangles). Upon phage infection, a sequence of the invading DNA (protospacer) is incorporated into the CRISPR array by the Cas1-Cas2 complex. The CRISPR array is then transcribed into a long precursor CRISPR RNA (pre-crRNA). In the CRISPR-Cas9 system, crRNA maturation requires tracrRNA, RNase III, and Cas9. The Cas9 protein contains two nuclease domains, the RuvC domain, and the HNH domain. Cas9 is guided by a sgRNA to induce a double-strand DNA break at a desired genomic locus. The sgRNA is composed of tracrRNA and crRNA. The tracrRNA hybridizes to the crRNA and binds to the Cas9 protein, forming the CRISPR-CAs9/sgRNA complex to edit genome sequences. DNA damage can be repaired by nonhomologous end joining (NHEJ), yielding short random insertions or deletions at the target site. Alternatively, a DNA sequence that shows partial complementarity to the target site can be inserted during homology-directed repair (HDR) for precise genome editing purposes.

Based on the current classification put forward by Makarova, the CRISPR-Cas system is classified into two classes [25]. Class 1 CRISPR-Cas systems include types Ⅰ, Ⅲ, and Ⅳ, and Class 2 includes types Ⅱ, Ⅴ, and Ⅵ, which all contain multiple Cas proteins that function as effector proteins that are responsible for pre-crRNA processing [26]. The CRISPR-Cas type II system is an ideal choice for gene editing because all domains essential for DNA cleavage are integrated into a single protein [26,27]. Among the type II systems, the CRISPR-Cas9 system (Figure 1) has been widely applied in targeting virulence genes and specific genes that encode antibiotic resistance in bacteria [28,29]. In the CRISPR-Cas9 system, an additional small noncoding RNA, called the trans-activating crRNA (tracrRNA), is indispensable to form a unique dual RNA hybridization via base pairs complementary with the repeat sequence in the crRNA. The crRNA and tracrRNA complex is called single-guide RNA (sgRNA). sgRNA binds to Cas9 and directs it to the target site to generate double-strand breaks in chromosomal DNA. In a recent study, truncated sgRNA greatly led to a 10-fold reduction of gene knockout frequency, which are relevant for future sgRNA design approaches and studies of Cas9-DNA interactions [30]. The Cas9 endonuclease consists of two domains named the HNH and RuvC domains. The HNH domain is responsible for cutting the complementary (target) DNA strand, which complements the crRNA guide. Its active state formation and stability can be maintained in the presence of catalytic Mg^2+^ [31]. The RuvC domain is involved in the cleavage of a non-complementary (nontarget) DNA strand [32]. 

Therefore, by designing multiple sgRNAs, it is facile and fast to use the CRISPR-Cas9 system to delete or insert specific sequences at the site of a genomic locus of interest in an extremely precise manner. The CRISPR-Cas9 system has received extensive attention for its extraordinary ability in genome editing and promising applications, including treating genetic diseases, genome engineering of bacteria, plants, and mammalian cells, and the reversal of antibiotic resistance as well [33]. The CRISPR-Cas system has also been successfully used in pathogenic fungi such as *Candida albicans*, *Aspergillus*, and *Cryptococcus* [34].

## 3. CRISPR-Cas System and Antibiotic Resistance

### 3.1. Relationships between the CRISPR-Cas System and Antibiotic Resistance

**Figure 2:**
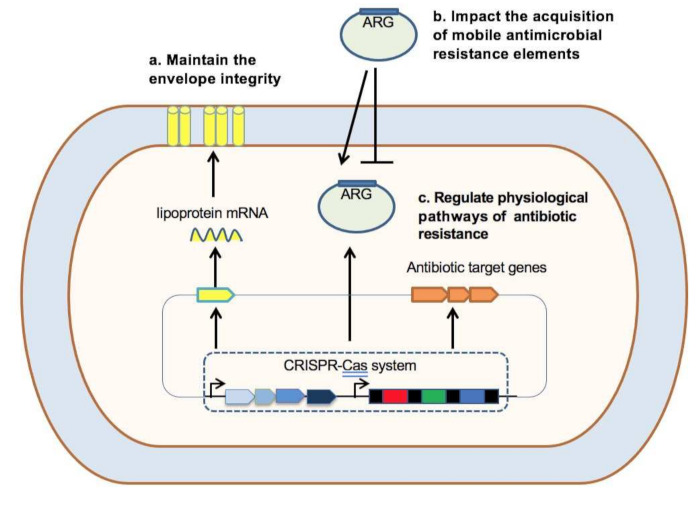
Relationships between the CRISPR-Cas system and antibiotic resistance in bacteria. a. The CRISPR-Cas system can maintain envelope integrity by regulating envelope lipoprotein expression to enhance antibiotic resistance, such as in *Francisella novicida*. b. The CRISPR-Cas system can impact the acquisition of mobile antimicrobial resistance elements to avoid antibiotic resistance, such as in *Campylobacter jejuni* and *Enterococcus faecalis*. c. The CRISPR-Cas system can regulate the physiological pathway involved in the antimicrobial resistance of bacteria, such as in *Klebsiella pneumoniae*. ARG: antibiotic resistance gene.

There are several studies indicate that the CRISPR-Cas system is associated with antibiotic resistance (Figure 2). For example, the Type I-F CRISPR system in *E. coli* was found associated with antibiotic susceptibility [35]. The CRISPR-Cas system in *Francisella novicida* maintains envelope integrity by regulating envelope lipoprotein expression to enhance antibiotic resistance [36]. The CRISPR-Cas system in *Campylobacter jejuni* was found involved in enhancing antibiotic resistance, as the deletion of the cas9 gene increased the sensitivity to antibiotics [37]. The findings of different studies reveal that the CRISPR-Cas system confers the competitive advantage over other variants for a population of bacteria that can acquire resistance genes [29]. In addition, the presence of the CRISPR-Cas system was correlated with the acquisition of antibiotic-resistant genes (ARGs), which can be positive or negative [38]: CRISPR-Cas-mediated immunity provides protection against foreign nucleic acids as well as the ARGs transfer. However, under a strong selective pressure imposed by antibiotics, the CRISPR-Cas system may be lost or devoid of function as they impede the acquisition of ARGs by horizontal gene transfer (HGT) [39]. 

For example, some studies have reported that the CRISPR-Cas system can prevent the acquisition of antibiotic resistance genes in bacteria. Mackow et al. found that *Klebsiella pneumoniae* harboring the CRISPR-Cas system displayed a high sensitivity to carbapenems, a kind of antibiotic resistance usually caused by *bla*_KPC_ plasmids [40]. In another study, Price and colleagues proved that the loss of the *cas9* gene facilitated *Enterococcus faecalis* acquisition of resistance genes through conjugation [41]. Besides that, CRISPR-Cas expression increased the sensitivity of *Mycobacterium smegmatis* to the environmental stress, including acidic and oxidative stress, as well as multiple anti-tuberculosis agents, through reducing the drug-induced persistence [42]. These data support that the CRISPR-Cas system can impact the acquisition of mobile antimicrobial resistance elements or regulate the physiological pathway involved in the antimicrobial resistance of bacteria.

### 3.2. CRISPR-Cas System-Based Genome Editing to Combat Bacterial Infection

**Figure 3:**
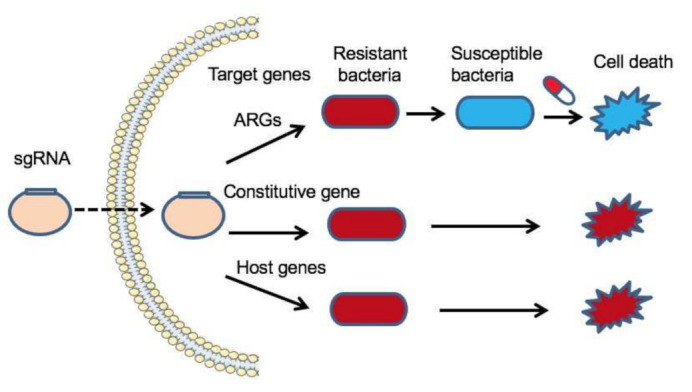
CRISPR-Cas system-based genome editing to combat bacterial infection. When the target genes are ARGs, the CRISPR-Cas system can restore the sensitivity of bacteria to antibiotics, and then the drugs will kill the susceptible bacteria. When the target genes are constitutive genes of the specific resistant bacteria, then the resistant bacteria will be killed. When the target genes are host chromosomal DNA that is cytotoxic to the resistant bacteria, the target cell will die because of the excision of the genome.

According to the CRISPR-Cas gene editing principle, the RNA-based spacer directs Cas proteins to target and cleave DNA that complements the spacers. In other words, guide RNAs can be designed to target virulence or antibiotic resistance genes that are specific to antimicrobial-resistant bacteria. Thus, the CRISPR-Cas9 system can be employed to neutralize antibiotic resistance genes in the targeted bacterial population without killing the beneficial bacteria in wild-type populations [43,44,45,46] (Figure 3). For example, the CRISPR-Cas9 system is being developed to restore the sensitivity to antibiotics in extend-spectrum beta-lactamase (ESBL)-producing *Escherichia coli* by identifying a conserved target sequence in >1000 ESBL mutants [44]. Moreover, with the high specificity of the CRISPR-Cas system, resistant strains can be selectively removed from complex bacterial populations by transforming the population with a plasmid or phage carrying a programmed CRISPR-Cas9 system targeting a unique sequence that only exists in resistant strains [43,47]. In *Staphylococcus aureus*, phagemid-mediated delivery of CRISPR-Cas9 was used to eliminate virulence genes and antibiotic resistance genes, thereby resensitizing bacteria to antibiotics [43]. In a recent study, Rodrigues et al. engineered conjugative plasmid pPD1 with a complete, constitutively expressed CRISPR-Cas9 targeting cassette that efficiently transfers to *Enterococcus faecalis* for the selective removal of *ermB* (encoding erythromycin resistance) and *tetM* (encoding tetracycline resistance) [46]. In vivo results showed that these transformants significantly reduced the prevalence of antibiotic-resistant intestinal *E. faecalis* and are immune to the uptake of antibiotic resistance determinants. The CRISPR-Cas9 system was also reported to cleave epidemic carbapenem-resistant plasmids, such as *bla*_KPC_-harboring IncFIIK-pKpQIL, IncN pKp58_N, and *bla*_NDM_-harboring IncX3 plasmids, through disrupting the partition gene *parA* in *K. pneumoniae* [48]. To increase the selective advantage of resensitized bacteria, Yosef et al. used temperate and lytic phages to deliver a programmed CRISPR-Cas9 system to destroy plasmids carrying beta-lactamase resistance genes *bla*_NDM-1_ and *bla*_CTX-M-15_ in *E. coli* [47], in which the CRISPR-Cas system targeted antimicrobial resistance genes was carried by a temperate phage. Strains transfected with this recombinant phage acquired resistance to lytic phages and thus had a selective advantage over resistant strains when treated with the same type of phage. Instead of directly killing bacteria, it aims to make bacteria sensitive to antibiotics and thereafter kill non-sensitized bacteria with lytic phages. This kind of strategy also eliminates the horizontal transfer of antibiotic resistance genes between bacteria. Apart from the CRISPR-Cas9, Kiga et al. developed CRISPR-Cas13a-based antimicrobial system in a bacteriophage capsid that is capable of effectively killing carbapenem-resistant *E. coli* and methicillin-resistant *S. aureus* targeting to sequence-specific antimicrobial resistance genes [49].

Furthermore, the CRISPR-Cas system can be used as a novel antimicrobial agent. Some reports have shown that the occasional or intentional acquisition of host chromosomal DNA by the CRISPR-Cas system is cytotoxic, which can result in cell death because of the excision of the genome [48,50]. Most of the CRISPR spacers match phages and plasmids, but some show similarity with chromosomal sequences, such as prophages, other mobile elements, and even the core genome. For example, Gomaa and colleagues reprogrammed the *E. coli* CRISPR-Cas type Ⅰ system with the spacer complementary to the essential *ftsA* gene, a critical gene involved in cell division, to kill bacteria efficiently regardless of genomic location, strand, or transcription activity [51]. Cañez et al. found that Type Ⅰ-E- and Type Ⅱ-A-based plasmids self-targeting *lacZ* genes were lethal in *Streptococcus thermophilus* survivors who had large genomic deletions during genome repair by homologous recombination [52].

The high efficiency of the CRISPR-Cas system in killing bacteria is attributed to Cas nuclease. For example, the introduction of self-targeting type Ⅰ systems in which Cas3 nuclease possesses both single-strand DNA exonuclease and 3′-5′ helicase activity induced rapid cell death and degraded the DNA away from the region of the targeted sequence region [53,54]. In another study, Hamilton et al. adopted the IncP type conjugative plasmid RK2 to deliver the CRISPR Cas9 system *E. coli* to *Salmonella enterica* with a high conjugation frequency to efficiently kill *S. enterica* [55]. The *cis*-conjugative plasmid was constructed based on pTA-Mob, a broad-host-range mobilization plasmid that is suitable for transferring large DNA to a large number of bacterial strains as donors by inserting the origin of transfer (oriT) and CRISPR system into it [56]. This plasmid had high conjugative efficiency because transconjugants became new donors for subsequent reconjugant. Therefore, the CRISPR-Cas9 system carried by this kind of plasmid kills *S. enterica* more efficiently. In *Mycobacterium tuberculosis*, an endogenous subtype III-A CRISPR-Cas system acts as an antimicrobial by introducing a recombinant phagemid carrying spacer-targeted essential genes into *M. tuberculosis* [57,58]. Moreover, the anti-tuberculosis bacteriophage can be delivered by inhalation devices, which implies versatile ways of drug delivery when using the CRISPR system to combat antibiotic-resistant strains [59]. 

These studies demonstrated that the CRISPR-Cas system can discriminate targeted strains better than antibiotics because even minor sequence differences are enough for the CRISPR-Cas system to identify. It is easy to realize that researchers can selectively eliminate closely related bacterial strains, whether in pure or mixed cultures, as long as they acquired the genome sequence information.

## 4. The Applications of Nanoparticles in Antibiotic Therapy

### 4.1. Nanoparticles Act as Antibacterial Materials

Nanoparticles (i.e., with at least one dimension between 1 nm and 100 nm) are widely used to enhance the delivery of antimicrobial agents act as novel antimicrobial material that is distinct from traditional drugs [60,61]. Nanoparticles (NPs) mainly rely on two mechanisms to act as promising antimicrobial agents against bacteria: (i) disruption of membrane potential and integrity and (ii) induction of oxidative stress via reactive oxygen species (ROS) generation catalyzed by NPs [62,63,64]. These two types of mechanisms can occur independently or simultaneously. 

Inorganic nanoparticles usually possess a high surface-area-to-volume ratio and unique physical and chemical properties, which enhance their antibacterial activity. For instance, silver nanoparticles (AgNPs) have been widely used as potent antimicrobials against bacteria because of their remarkable bactericidal effect exceeding that of other metal oxides and reduced possibility of inducing resistance. Gold nanoparticles (AuNPs) are another type of inorganic nanoparticles that widely tested as antimicrobial agent because of their photothermal activity, biocompatibility, and easy modification with small antimicrobial drugs [65,66,67,68]. Furthermore, there is a particular interest in biosynthesized nanoparticles, such as copper nanoparticles prepared with *Zingiber officinale,* and *Curcuma longa* that displayed a remarkable antibacterial activity against multidrug resistant *Staphylococcus aeureus* [68]. Metal oxide NPs of titanium dioxide (TiO_2_) that adopts a similar bactericidal mechanism as AgNPs to kill both Gram-positive and Gram-negative bacteria were extensively reported [69]. Moreover, combined with other nanomaterials, such as zinc oxide (ZnO), TiO_2_ nanoparticles showed considerable activity against methicillin-resistant *Staphylococcus aureus* (MRSA) [70]. Compared with the metal oxides described above, antimicrobial ZnO is much safer and holds high biocompatibility with human cells [71,72,73,74]. ZnO NPs dispersed in ionic liquids displayed a high efficiency in killing the skin-specific bacterium *Staphylococcus epidermidis* through ROS production, resulting in bacterial cell lysis without toxicity to normal keratinocyte cells [72].

Besides inorganic nanoparticles, polymeric nanoparticles are used in antibacterial applications as well. Polymeric nanoparticles can directly kill microbes by interacting with bacterial cell walls, which are typically negatively charged. Polymeric nanoparticles are endowed with intrinsic antimicrobial activity by incorporating cationic and hydrophobic moieties into polymer chains, such as quaternary ammonium groups, alkyl pyridiniums, and phosphonium. Cationic groups are able to disrupt the cell membrane; meanwhile hydrophobic moieties help to penetrate and burst into the membrane. It has been reported that the antibacterial effect can be improved by increasing the density of cationic charges, which enhance the electrostatic interactions with anionic membranes [75]. Therefore, it is possible to design various polymeric nanoparticles with a positively charged surface to create different antimicrobial materials. For instance, Takahashi et al. reported a cationic amphiphilic polymeric nanoparticle that effectively killed the planktonic cariogenic bacterial *Streptococcus* mutants and prevented biofilm formation [76]. Another antibacterial peptide-based copolymer micelle was synthesized and exhibited potent bactericidal efficacy against both gram-negative and gram-positive bacteria. Transmission electron microscopy (TEM) results revealed that the micelles can penetrate and then destroy the bacterial cell membrane, leading to cell lysis [77]. Due to their prominent bactericidal properties, NPs were also applied in the coating of human implantable devices, wound dressings, bone cement, and dental materials [77,78,79,80,81]. 

### 4.2. Nanoparticles for Drug/Gene Delivery to Combat Bacterial Infection

Apart from directly eliminating bacteria, polymeric nanoparticles can indirectly fight bacteria by acting as drug carriers to deliver antibiotics, antimicrobial peptides, and antimicrobial agents to the target parts of the body. The main limitations of clinical antibiotic therapy are the low drug bioavailability, poor penetration to bacterial infection sites, side effects of antibiotics, and also antibiotic resistance [82]. Polymeric nanoparticles can protect antibiotics from being environmentally deactivated and improve their pharmacokinetics and distribution in the body. The most prevalent approach to produce antibiotic-loaded polymeric nanoparticles is entrapping antibiotics into polymeric particles, which enhances the solubility of hydrophobic drugs and enhances the antibacterial effect. In a study, ciprofloxacin-loaded polymeric nanoparticles were developed with a continuous moderate release rate and high antibiotic concentration at the targeted site [83]. Hasan and coworkers synthesized positively charged clindamycin-loaded polyethyleneimine nanoparticles (Cly/PPNPs) and proved that they had enhanced bactericidal efficacy against MRSA because of their strong bacterial adhesive ability [84]. In another recent study, polymeric nanoparticles loaded with the broad-spectrum antibiotic sparfloxacin and the anti-inflammatory immunosuppressant tacrolimus displayed potent antibacterial activity and the ability to precisely locate inflammatory cells to treat acute lung sepsis [85]. These results showed that polymeric nanoparticles have promising prospects in preventing or treating infectious diseases.

In addition to drug delivery, nanoparticles for gene delivery have also attracted enormous attention. Naked genetic elements cannot efficiently enter target cells because of adsorption of serum proteins, rapid clearance in blood circulation, phagocyte uptake, incapability of endosomal escape, lack of targeting ability, and toxicity induced by the immune system. To overcome these drawbacks, many types of nanoparticles have been developed as gene carriers, including polymeric nanoparticles, lipid nanoparticles, and metal nanoparticles [86]. For instance, DNA or RNA can be encapsulated in poly (lactic-coglycolic acid) nanoparticles to protect them from degradation during circulation. Among the diverse nanoparticles, lipid-based nanoparticles have been extensively explored because of their liposomal-like characteristics, which facilitate cellular entry. To improve the targeting ability, ligands such as antibodies, proteins, or peptides, can be functionalized with the gene-delivery nanoparticles to specifically bind to the receptors on the targeted cells [87,88]. Polyethyleneglycol (PEG) modification with nanoparticles is widely used to inhibit nonspecific interactions with serum proteins and increase active targeting efficiency by increasing their circulation time in the bloodstream [89,90,91]. 

## 5. Nanoparticles as CRISPR-Cas9 Delivery Systems

### 5.1. Delivery Strategies for CRISPR-Cas9 System

**Figure 4:**
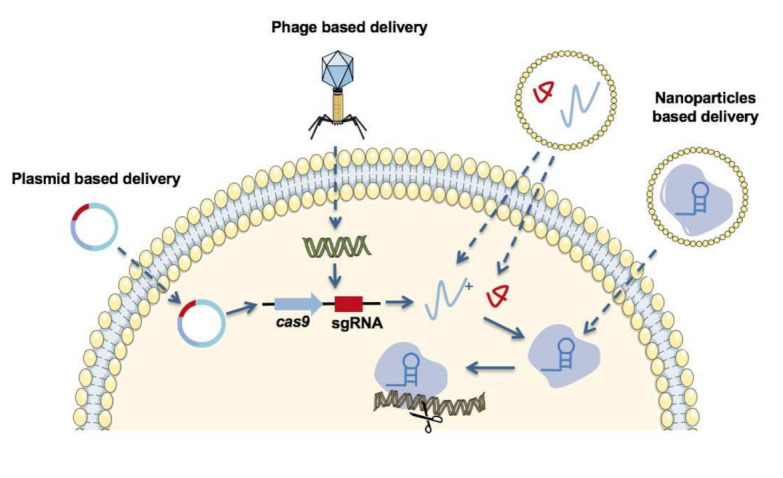
Different strategies for CRISPR-Cas9 system delivery to edit genes. Plasmid-based delivery: The plasmid-borne CRISPR-Cas system can be transferred into cells and transcribed into Cas9 mRNA and sgRNA. Cas9 mRNA is translated into the Cas9 protein, which forms a ribonucleoprotein (RNP) complex with sgRNA. Then, the RNP complex edits the target genes directed by sgRNA. Phage-based delivery: CRISPR-Cas system coding sequences are delivered by phages into cells. Nanoparticle-based delivery: Cas9 and sgRNA can be delivered either in the form of mRNA or Cas9-sgRNA ribonucleoprotein (RNP) complexes with the help of nanoparticles.

To apply CRISPR-Cas9 system gene editing, the endonuclease Cas9 and guide RNA for the CRISPR system should be included in delivery vehicles. Strategies for delivery of the CRISPR/Cas9 system (Figure 4) include: (i) a plasmid encoding Cas9 and guide RNA; (ii) two separate plasmids encoding Cas9 and guide RNA; (iii) mRNA for Cas9 and guide RNA; and (iv) a Cas9-guide RNA complex [92]. Compared with the mRNA delivery form, a plasmid encoding Cas9 and guide RNA is more stable and cost-effective than the mRNA forms of Cas9 and guide RNA. However, it is difficult for a CRISPR-delivered DNA plasmid to enter the cellular and nuclear membranes and then take effect. The large size of the plasmid, which consists of many noncoding sequences, makes it challenging to be encapsulated with nanoparticles. However, the constitutive expression of CRISPR in mammalian cells is prone to increase off-target effects and the risk of insertion mutagenesis by randomly integrating plasmid DNA into the host genome [93].

Encoded Cas9 mRNA delivery can overcome these drawbacks and be directly translated in the cytoplasm without entering the nucleus, resulting in reduced off-target effects and less risk of integration into the genome. However, mRNA is unstable and easily degraded by RNases either during the synthesis or when applied in vivo. Cas9-guide RNA ribonucleoprotein (RNP) complex delivery is the most straightforward and rapid approach for gene editing and has fewer off-target effects as well as low immunogenicity [94]. However, it is also the most challenging approach because of the large size of the Cas9 protein, the super negative charges of guide RNA, and the vulnerability to degradation and denaturation of RNPs during the entire process [95,96,97]. The development of stable and reliable nanoparticles for RNP delivery systems remains elusive. Plasmids or viral vectors, such as species-specific phages and adeno-associated viruses, are exploited for CRISPR-Cas delivery [98,99]. However, both methods are limited in practical use because of their low loading and packaging efficiency, narrow host range, risk of carcinogenesis, and immunogenicity.

### 5.2. Nanoparticles for CRISPR-Cas9 Delivery

**Figure 5:**
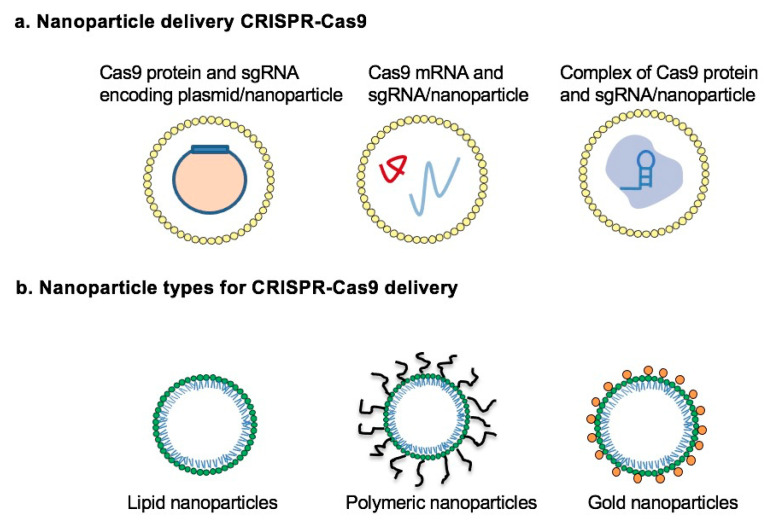
Nanoparticles for drug/gene delivery to combat bacterial infection. (**a**) Nanoparticles enfold different CRISPR-Cas9 delivery forms: Cas9 protein and sgRNA encoding plasmid, Cas9 mRNA and sgRNA, Complex of Cas9 protein and sgRNA. (**b**) Nanoparticles for CRISPR-Cas9 delivery: lipid nanoparticles, polymeric nanoparticles, and gold nanoparticles.

To overcome the drawbacks of viral vectors, nonviral vectors for delivering the CRISPR system, mainly through nanoparticle-based deliveries such as lipid nanoparticles, polymeric nanoparticles, and gold nanoparticles (AuNPs), have attracted significant interest in researchers [100] (Figure 5). Nanoparticles with chemical modification can protect Cas9 mRNA by improving its stability. Lipid nanoparticles are one of the most extensively explored nanoparticle systems for CRISPR delivery. Several lipid-based carriers for gene therapy have been approved for clinical trials [101]. Lipid nanoparticles are amphiphilic compounds that help encapsulate negatively charged CRISPR plasmid DNA and mRNA, guiding and protect RNA from crossing the cell membrane. Liu and coworkers reported that BAMEA-O16B, a lipid nanoparticle integrated with disulfide bonds, delivered Cas9 mRNA and sgRNA simultaneously to knock out green fluorescent protein (GFP) expression in human embryonic kidney cells [102]. This gene knockout efficiency can be as high as 90%. However, when designing nanoparticles for gene delivery, it is quite challenging to selectively target specific tissues. Cheng et al. developed a strategy named selective organ targeting (SORT), which was used to modify lipid NPs with a diverse percentage of SORT molecules to precisely deliver Cas9 mRNA/sgRNA together with Cas9 ribonucleoprotein to the liver, lung, and spleen [103]. To enhance the genome editing efficiency and cell-selective ability, Tang et al. designed a cationic lipid system that includes a phenylboronic acid (PBA) group to self-assemble with CRISPR/Cas9 mRNA into nanoparticles. This kind of configuration showed an improved cellular uptake by cancer cells that overexpress surface sialic acid (SA), due to the interfacial interaction of PBA and SA [104]. This delivery system suppressed p53 mRNA significantly in Hela cancer cells with a higher efficiency than non-cancer cells. Similarly, liposome-templated hydrogel nanoparticles (LHNPs) for CRISPR/Cas9 delivery were synthesized to knock out polo-like kinase1 (PLK1) gene in a mouse flank tumor model to inhibit tumor growth with higher efficiency than commercial agent Lipofectamine 2000 [105]. Several nanoparticles delivering the CRISPR-Cas9 system are compiled in Table 1.

Polymeric NPs are another important method for CRISPR delivery owing to their low immunogenicity and high biocompatibility. Polymeric nanoparticles can be conjugated with cell-penetrating peptides on the surface for quick cellular uptake and/or nuclear localization signal peptides for delivery inside cells. Carboxylated branched poly (β-amino ester) nanoparticles were reported to be an efficient way to deliver Cas9 RNPs for increased hydrogen bonding and hydrophobic effects [118]. The authors proved that this Cas9 RNP delivery polymer induced high gene editing levels both in vitro and in vivo at relatively low RNP doses. Apart from Cas9 RNPs, they also found that polymeric nanoparticles are applicable for transferring CRISPR-Cas9 plasmids [118]. Recently, Nguyen et al. reported a novel platform combining polymeric nanoparticles and a modified homology-directed repair template [124]. Polymeric NPs consisted of anionic poly-L-glutamic acid (PGA) help stabilize RNPs by shielding excess positively charged residues of the Cas9 protein, which resulted in enhancing gene editing efficiency and cell viability and reducing off-target effects. The polymeric and hybrid poly-L-arginine hydrochloride/dextran sulfate (PARG/DEXS)_3_ capsules that deliver CRISPR/Cas9 in the form of plasmids was reported to successfully knock out the dTomato gene in HEK293 T cells [116]. In addition, a previous research study has demonstrated the macrophage-specific gene editing using CRISPR/Cas9 components delivered through cationic lipid-assisted PEG-b-PLGA nanoparticles (CLANs), and PEGylation was proposed as an effective strategy to prevent non-specific interactions and avoid immune recognition [117]. 

In addition to polymeric NPs, AuNPs are considered quite suitable for CRISPR RNP complex delivery due to their unique controllable features, precise modification, and relative safety compared to lipid and polymer nanocarriers. Shahbazi and coworkers designed an AuNP-based CRISPR nanoformulation (AuNP/CRISPR) with the conjugation of the CRISPR RNP complex on the surface of AuNPs [113]. This AuNP/CRISPR delivery system successfully penetrated hard-to-transfect CD34+ hematopoietic cells (HSPCs) and edited CCR5 and γ-globin promoter gene loci without generating any adverse effects. Their results showed that other blood cell types could also be edited by AuNP/CRISPR, which provides a powerful delivery method in different cells. Moreover, another study proved that the cationic HIV-1-transactivating transcriptor (TAT) peptide-modified gold nanoclusters-carrying CRISPR/Cas9 system could be used to decrease lipoprotein cholesterol (LDL-C) level by disrupting the *Pcsk9* gene in mice model, which indicates a new therapeutic approach for the treatment of cardiovascular disease [114]. Besides that, gold nanoclusters (AuNCs) were also used to deliver *Streptococcus pyogenes* Cas9 endonuclease (SpCas9) into the nucleus via a highly pH-dependent assembly process [125]. The SpCas9-AuNC complex is stable at higher pH and disassembles at lower pH. The authors successfully employed this delivery system to knock out the E6 oncogene responsible for cervical cancer malignant transformation. Although there are few studies on applying NP/CRISPR systems to bacteria, similar targeted strategies can be adopted to inhibit microbial infections by directly eradicating pathogens or removing antimicrobial resistance genes [15,119]. For example, Kang et al. reported a nanosized CRISPR complex that consisted of a polymer-derivatized Cas9 endonuclease and a single guide RNA targeting the major methicillin resistance gene *mecA* in *S. aureus*. The polymer-derivatized Cas9 endonuclease was produced by covalent modification with branched polyethyleneimine. This nanosized CRISPR complex can be successfully delivered into MRSA and efficiently edit the bacterial genome, resulting in reduced growth of MRSA [119].

## 6. Summary and Future Prospects

CRISPR-Cas systems are promising gene-editing tools for controlling the prevalence of antibiotic resistance genes among bacteria and eliminating pathogens with high precision. Off-target effects, high cost, systemic delivery, as well as delivery efficiency are major challenges in this regard. These issues may be overcome via the employment of nanomaterials as nonviral carriers for the delivery of the CRISPR/Cas9 system. Multiple innovative nanoparticles of polymers, lipids, and gold have been developed. Although tremendous progress has been made in designing these nanoparticles to optimize the effect of the CRISPR-Cas system, achieving higher efficiency and safer delivery of the system remains a challenge, and further investigations are needed.

Efficient packaging and localization of the CRISPR-Cas components are two main obstacles for NP/CRISPR application. As we discussed above, NPs can be tailored in diverse ways. PEGylation modification of the surface of the system is a common strategy to reduce reticuloendothelial system (RES)-mediated clearance and increase duration time in the blood or tissue. Other modifications, such as cell-penetrating peptides and specific cell receptors, can improve cell internalization and interaction with targeted cells. Immunogenicity and off-target editing effects are also concerns regarding in vivo applications. Nevertheless, the integration of nanoparticles and the CRISPR system is still in the early stage. There is a long way to go before the successful application of engineered nanoparticles and CRISPR-Cas systems to treat bacterial infections and control the dissemination of antimicrobial-resistant bacteria.

## Figures and Tables

**Table 1:** Nanoparticle delivery systems for CRISPR-Cas9.

Delivery System	Crispr-Cas Form	Study Objective	Target Gene	In Vitro/ Vivo	Brief Result	Ref
Lipid-based NPs.	Cas9 mRNA and sgRNA	Hepatocytes, C57BL/6 mice	PCSK9	In vivo	The lipid NPs delivered-CRISPR/Cas9 effectively knocked the protein level of PCSK9 in mouse serum down to 20%.	[99]
Lipid-based NPs	Cas9 mRNA and sgRNA	C57BL/6 mice	PTEN PCSK9	In vivo	SORT LNPs mediated effective tissue-specific genes PTEN and PCSK9 editing in the liver.	[100]
Lipid-based NPs	Cas9 mRNA and sgRNA	Hela cells	GFP HPV18E6	In vitro	treatment of HeLa cells with PBA-BADP/Cas9 mRNA/sgHPV18E6 NPs showed GFP knocked out efficiency up to 50% and resulted in 18.7% indel of HPV18E6 gene.	[101]
Lipid-based NPs	Ribonucleoproteins	U87 cells Mice bearing tumor	PLK1	Both in vitro and in vivo	LHNPs co-encapsulated with Cas9 and minicircle sgRNA were capable of efficiently inhibiting PLK1 expression to 36.3% and inhibit tumor growth	[102]
Lipid-based NPs	Cas9 mRNA and sgRNA	BMDMs C57BL/6 mice	NLRP3	Both in vitro and in vivo	Disrupt NLRP3 of macrophages in vitro by CLANmCas9 with an efficiency rate of 70.2%, compared to the rate of 58.6% in vivo.	[106]
Lipid-based NPs	Cas9 mRNA and sgRNA	Splenic endothelial cells HEK293	ICAM-2	In vivo	LNPs can edit endothelial cells successfully, and the ideal Cas9: sgRNA ratio will depend on the relative stability of the two molecules.	[107]
Lipid-based NPs	Cas9 mRNA and sgRNA	HEK293, GBM 005 cells	GFP, PLK1	In vivo	CRISPR-LNPs against PLK1 enabled up to ~70% gene editing in vivo, inhibited tumor growth by 50%, and improved survival by 30%.	[108]
Lipid-based NPs	Ribonucleoproteins	B16F10 cells	PD-L1	In vitro	VLN-sgPD-L1 reduced the expression of PD-L1 to 41.3% and thus suppressed tumor growth in vivo.	[109]
LNP-INT01	Cas9 mRNA and sgRNA	Cd-a-mice	TTR	In vivo	CRISPR-LNPs against TTR in the liver of mice resulted in a 97% reduction in serum protein levels that persisted for at least 12 months.	[110]
Lipidoid NPs	Ribonucleoproteins	Hela-DsRed cells	GFP	In vivo	LNPs-based CRISPR/Cas9 system displayed high GFP knockout efficacies ~70% with low cytotoxicities.	[58]
Gold/lipid NPs	Plasmid DNA	Melanoma	Plk-1	Both in vitro and in vivo	AuNPs-based CRISPR/Cas9 system led to about 65% down-regulation of Plk-1 protein triggered by the photothermal effect.	[111]
Gold NPs	Ribonucleoproteins	Fragile X syndrome	mGluR5	In vivo	CRISPR-Gold targeting the mGluR5 gene reduced the protein level by 40–50% in the mouse models that have fragile X syndrome	[112]
Au NPs	Ribonucleoproteins	HSPCs	CCR5	In vitro	NPs-mediated CRISPR/Cas9 successfully penetrated into HSPCs and produced up to 17.6% total editing.	[113]
Au NPs	Ribonucleoproteins	Hepa 1-6 cells mice	Pcsk9	Both in vitro and in vivo	This Au Nps-based CRISPR/Cas9 delivery system induced significant Pcsk9 editing in vitro and reduced the LDL-C level to 30% compared with the control group by knocking out the Pcsk9 gene in mice.	[114]
polymeric NPs	Plasmid DNA	Chronic myeloid leukemia	CML-related BCR-ABL	Both in vitro and in vivo	CLANpCas9/gBCR-ABL disrupted the BCR-ABL gene in vitro with an efficiency rate of 46.8%, reduced the mRNA level to 41.9%, and greatly inhibited the protein expression of BCR-ABL in CML mice.	[115]
polymeric NPs	Plasmid DNA	HEK293T cell	dTomato	in vitro	Polymeric microcarriers for CRISPR/Cas9 displayed high gene knockout efficiency up to 70% in the transfected cells.	[116]
polymeric NPs	Plasmid DNA	HFD-induced T2D mice	NE	In vivo	CLANpCas9/gNE targeting the neutrophil elastase (NE) gene effectively disrupted the NE gene in the mouse have type 2 diabetes (T2D) with the gene knock-out rate of 26.4% and mitigated the insulin resistance by reducing neutrophils-related inflammation	[117]
polymeric NPs	Plasmid DNA	Hela cells HEK293T cell	GFP iRFP	In vitro	A novel reporter system involving PBAEs-CRISPR carrier for easy detection of gene knockout at one and two genomic sites	[118]
Polymeric NPs	Ribonucleoproteins	S. aureus	MecA	In vitro	Cr-Nanocomplex treatment resulted in a significant inhibition in MRSA growth in the presence of methicillin by disrupting the mecA gene.	[119]
Core-shell NPs with iron oxide core and PEI coating	Plasmid DNA	Porcine fetal fibroblasts	H11	In vitro	Magnetic NPs carrying CRISPR/Cas9 displayed 3.5 times higher efficiency compared to the classic lipofection method	[120]
PEI magnetic NPs	Plasmid DNA	HEK293 cell	TLR-3	In vitro	Magnetic NPs-CRISPR/Cas delivery system enabled site-specific incision with the combination of an inhomogeneous magnetic field.	[121]
PLGA NPs	Ribonucleoproteins	HSPCs	γ-globin gene	In vitro	CRISPR/Cas9-PLGA-NPs-mediated gene inaction ofγ-globin gene in HSPCs led to the increase in the HbF expression (51.7%) in a concentration-dependent manner.	[122]
pH-responsive polymeric NPs	Plasmid DNA	B16F10 cells	Cdk5	Both in vitro and in vivo	The CRISPR/Cas9 encapsulated in nanoparticles specifically knock out the Cyclin-dependent kinase 5 (Cdk5) gene to significantly attenuate the expression of PD-L1 on tumor cells.	[123]

## Data Availability

No new data were created or analyzed in this study.
